# Supplementary material for: Mutations in PCYT2 disrupt etherlipid biosynthesis and cause a complex hereditary spastic paraplegia
Source: Brain. 2019 Oct 22;142(11):3382–97. doi: 10.1093/brain/awz291 (PMC6821184; doi:10.1093/brain/awz291)
Supplement: awz291_Supplementary_Data [file awz291_supplementary_data.zip › awz291-Suppl_data/Supplementary_Data4.zip!/Supplementary information 4 - plasma lipidomics.zip!/Plasma lipidomics/Control vs Patient3b/Statistical plots and data/TG[O] species/Boxplots.pdf]

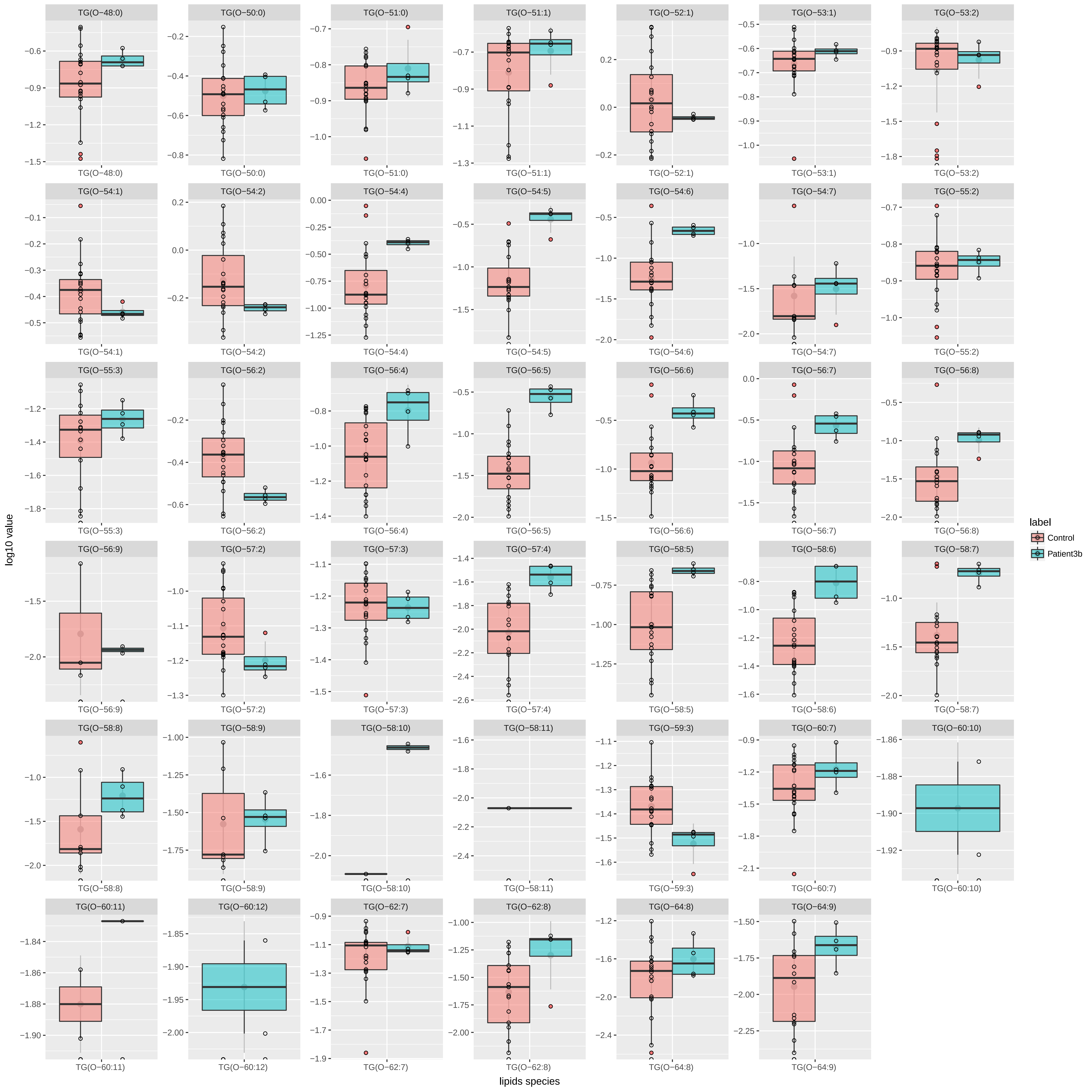

TG(O-48:0)
TG(O-50:0)
TG(O-51:0)
TG(O-51:1)
TG(O-52:1)
TG(O-53:1)
TG(O-53:2)
TG(O-54:1)
TG(O-54:2)
TG(O-54:4)
TG(O-54:5)
TG(O-54:6)
TG(O-54:7)
TG(O-55:2)
TG(O-55:3)
TG(O-56:2)
TG(O-56:4)
TG(O-56:5)
TG(O-56:6)
TG(O-56:7)
TG(O-56:8)
TG(O-56:9)
TG(O-57:2)
TG(O-57:3)
TG(O-57:4)
TG(O-58:5)
TG(O-58:6)
TG(O-58:7)
TG(O-58:8)
TG(O-58:9)
TG(O-58:10)
TG(O-58:11)
TG(O-59:3)
TG(O-60:7)
TG(O-60:10)
TG(O-60:11)
TG(O-60:12)
TG(O-62:7)
TG(O-62:8)
TG(O-64:8)
TG(O-64:9)
log10 value
lipids species
label
Control
Patient3b
